# Prognostic value of low-cost white blood cell indices and procalcitonin for mortality in Rwandan sepsis patients: a prospective intensive care unit study

**DOI:** 10.1186/s41182-025-00815-4

**Published:** 2025-10-09

**Authors:** Emmanuel Kundukundwe, Theodette Nizeyimana, Ayingeneye Grace Mutoni, Aline Muhimpundu, Enatha Mukantwari, Cedrick Izere, Solomon Ali, Araya Gebreyesus Wasihun, Tiruzer Bekele, Thaddee Nshimiyimana, Augustin Nzitakera, Ella Larissa Ndoricyimpaye, Schifra Uwamungu, Eliah Shema, Elizabeth Gori, Wossenseged Lemma, Cuthbert Musarurwa

**Affiliations:** 1 Department of Biomedical Laboratory Sciences, School of Health Sciences, College of Medicine and Health Sciences, University of Rwanda, P.O. Box 3286, Kigali, Rwanda; 2 St. Paul’s Hospital Millennium Medical College, Addis Ababa, Ethiopia; 3 Department of Medical Microbiology and Immunology, College of Health Sciences, Mekelle University, P.O. Box 1871, Mekelle, Tigray Ethiopia; 4 Tigray Health Research Institute, THRI, Mekelle, Tigray, Ethiopia; 5 Department of Pathology, University of Gondar, Gondar, Ethiopia; 6 Department of Medical Biochemistry, Molecular Biology and Genetics, School of Medicine and Pharmacy, College of Medicine and Health Sciences, University of Rwanda, Huye, Rwanda

**Keywords:** Sepsis, Intensive care unit, Neutrophils, Procalcitonin, White blood cells, Biomarkers, Prospective cohort, Survival analysis, ROC curves, AUC, Rwanda

## Abstract

**Background:**

In resource-limited settings, early identification of sepsis and low-cost mortality predictors is critical for intensive care unit (ICU) triage. This study evaluated the prognostic value of baseline sociodemographic factors, routine hematological indices, and serum procalcitonin (PCT) levels for 40-day mortality among adult ICU patients meeting Sepsis-2 criteria in Rwanda.

**Methods:**

A prospective cohort of 125 ICU patients was followed for 40 days. Baseline variables included sex, age, PCT, total white blood cell (WBC) count, differential counts (neutrophils, basophils, eosinophils, monocytes, lymphocytes), and neutrophil-to-lymphocyte ratio (NLR). Survival probabilities were estimated using Kaplan–Meier curves and log-rank tests. Cox proportional hazards models identified independent mortality predictors, with assumptions tested via Schoenfeld residuals and multicollinearity assessed using variance inflation factors. Time-dependent receiver operator curve (ROC) analysis evaluated model performance at days 6, 10, and 15 using the area under the curve (AUC) values.

**Results:**

Of 125 patients, 56 (44.8%) were female. Median age was 41 years for survivors and 50 years for non-survivors (*p* = 0.097). In multivariable Cox regression, elevated neutrophil counts were independently associated with increased mortality [adjusted hazard ratio (aHR)] 1.99; 95% CI (confidence intervals) 1.37–2.88; *p* < 0.001), corresponding to a twofold higher hazard of death for approximately a threefold increase in neutrophil count. No significant associations were found for sex, age, or PCT. ROC analysis showed that models integrating neutrophils and total WBC (TotalWBC) achieved the highest predictive accuracy, with AUCs ranging from ~68% to 71% across all time points, outperforming simpler models.

**Conclusions:**

Elevated neutrophil counts at ICU admission are independently associated with increased mortality. Integrating absolute neutrophil and WBC data into predictive models enhances early mortality risk stratification. These findings underscore the value of routine biomarkers and robust modeling to guide timely interventions in resource-constrained ICU settings.

**Supplementary Information:**

The online version contains supplementary material available at 10.1186/s41182-025-00815-4.

## Introduction

Sepsis remains a leading cause of mortality worldwide, posing a persistent challenge to global health due to its complex and rapidly evolving pathophysiology [1]. Sepsis is characterized by a dysregulated host immune response to infection, which can swiftly progress to life-threatening organ dysfunction [2]. Over the past three decades, efforts to standardize the diagnosis and management of sepsis have led to multiple clinical frameworks and scoring systems [3, 4]; yet early recognition and effective intervention continue to determine survival outcomes, especially in critically ill patients.

The burden of sepsis is disproportionately high in low- and middle-income countries, particularly across sub-Saharan Africa, where limitations in healthcare infrastructure compound its impact [5, 6]. Risk factors such as widespread antimicrobial resistance, high prevalence of parasitic and bacterial infections, human immunodeficiency virus (HIV) seropositivity, and tuberculosis increase disease complexity and obstruct timely clinical diagnosis [7]. Rwanda exemplifies these challenges, with only four hospitals equipped with intensive care units (ICU) facilities and a national total of 39 critical care beds. Like most other African countries, the data on sepsis epidemiology and diagnostic schemes are scarce. However, a retrospective study conducted at major referral hospitals in Rwanda revealed the sepsis mortality rate to be as high as 50% [8]. The combination of high care costs and limited access to advanced laboratory diagnostics further underscores the need for efficient, context-appropriate tools to support evidence-based sepsis management.

To improve early sepsis diagnosis and prognosis, over 250 biomarkers have been investigated globally [9]. Among these, procalcitonin (PCT) has emerged as a promising biomarker candidate, particularly for identifying bacterial infection and informing antibiotic stewardship [10, 11]. Meanwhile, white blood cell (WBC) indices, especially the neutrophil count and neutrophil-to-lymphocyte ratio (NLR), have gained recognition as practical inflammatory markers with prognostic relevance [12, 13]. Though PCT and NLR have been studied extensively in high-resource settings, their performance in African ICU environments, especially in predicting death, remains poorly characterized.

The present study aimed to address this knowledge gap by evaluating the prognostic value of PCT and WBC parameters in predicting mortality among adult patients with suspected sepsis admitted to two national referral hospitals in Rwanda. We hypothesized that higher baseline neutrophil counts, NLR, and PCT levels would be independently associated with an increased risk of all-cause mortality. By examining these biomarkers in a real-world, resource-limited clinical setting, this research sought to inform biomarker-guided triage strategies and contribute to the development of tailored diagnostic frameworks for sepsis management in sub-Saharan Africa.

## Methods

### Study design

This prospective cohort study was conducted in the ICUs of two major Rwandan referral hospitals: Butare University Teaching Hospital (CHUB) and Rwanda Military Referral and Teaching Hospital (RMRTH). These referral hospitals are among the four leading hospitals in Rwanda, with the largest ICUs. RMRTH is located in Kigali, providing healthcare services to approximately 2 million civilian and military patients and has 11 ICU beds for adults. On the other hand, CHUB, located in the southern province of Rwanda, serves around four million people and has seven ICU beds. The ICUs of both referral hospitals screen sepsis patients and provide intensive care services for the management of severe sepsis and septic shock, as defined under the Sepsis-2 framework. As Sepsis-3 criteria are not routinely applied in Rwandan practice, this study used SIRS-based Sepsis-2 criteria to enrol 125 consecutive adults (≥ 18 years) admitted with suspected sepsis at the study sites. The patients were followed up over 40 days post-ICU admission to assess survival outcomes. The 40 days were chosen to capture extended ICU stays frequently observed in Rwandan hospitals, which often exceed 28 days, while avoiding the dilution seen in 60-day endpoints. The study was conducted from January to April 2025 (4 months).

### Sample size determination

The sample size was calculated using the single-proportion formula, based on an estimated sepsis mortality prevalence of 50% in Rwandan ICU patients from prior retrospective studies [8]. Using a 95% confidence level and an 8% margin of error, the minimum required sample size was approximately 150 participants. However, due to logistic constraints, limited study duration, and early mortality leading to the exclusion of patients before biomarker sampling, the final sample size achieved was 125 participants.

### Inclusion and exclusion criteria

Sepsis was defined as a suspected or confirmed infection accompanied by a systemic inflammatory response, characterized by at least two of the following clinical criteria: body temperature > 38 °C or < 36 °C, heart rate > 90 beats per minute, respiratory rate > 20 breaths per minute or partial pressure of carbon dioxide < 32 mmHg, or WBC count > 12,000 cells/mm^3^ or < 4000 cells/mm^3^, or > 10% bands on differential count [14]. Patients who had commenced antimicrobial treatment within the preceding 48 h, those with preexisting conditions significantly affecting PCT levels (such as medullary thyroid carcinoma or small cell lung cancer), and immunocompromised patients (including those with advanced HIV/AIDS, receiving chemotherapy, or post-organ transplants) were excluded from the study.

### Outcome measure

The primary outcome measure was all-cause mortality within the ICU admission period. Mortality status was assessed prospectively during follow-up. Patients were classified as survivors or non-survivors based on vital status at day 40. Patients discharged alive before day 40 were followed up to day 40 and classified as survivors if alive. This time-to-event outcome was analyzed using survival analysis techniques, with time measured in days from ICU admission to death or censoring.

### Data collection

A total of 5 mL venous blood was collected by venipuncture from each participant within 24 h of ICU admission for laboratory analysis. The blood sample was split equally into two blood collection tubes: a plain red-top tube for PCT measurement and an EDTA tube for complete blood count (CBC) evaluation. For PCT measurement, the red-top tubes were centrifuged to harvest serum, which was analyzed using the electrochemiluminescence immunoassay method on the COBAS e 411 analyzer (Roche Diagnostics, Rotkreuz, Switzerland). The assay utilized a sandwich complex formed between serum PCT and two monoclonal PCT-specific antibodies, one biotinylated and the other labeled with a ruthenium complex. Streptavidin-coated microparticles facilitated the binding of the complex to the solid phase. The mixture was aspirated into the measuring cell, where magnetic capture of microparticles at the electrode enabled chemiluminescent emission detection through voltage application. The assay demonstrated linearity across a range of 0.02–100 ng/mL. Samples exceeding 100 ng/mL were manually diluted in a 1:4 ratio to ensure accurate quantification. CBC parameters including WBC, neutrophils, monocytes, basophils, eosinophils, and NRL were analyzed using the Sysmex XS-500i automated hematology analyzer (Kobe, Japan) in accordance with the manufacturer’s guidelines. All analyses adhered strictly to the principles of good clinical laboratory practice.

Sociodemographic data, including age and sex, and clinical data, such as admission date and overall clinical outcome, were collected using a structured questionnaire and extracted from patient medical records. Patients were followed up throughout the duration of their ICU stay, with outcomes including mortality and discharge status recorded. After ICU discharge, patients were followed up for up to 40 days to determine their survival status. Bias was minimized by consecutively enrolling all Sepsis-2—eligible patients; using standardized automated analyzers per manufacturer protocols; having trained staff blinded to outcomes collect and enter data; and enforcing data quality through double entry with periodic verification checks.

### Statistical analysis

Survival probabilities over the 40-day follow-up period were estimated using the Kaplan–Meier method.

Survival curves were constructed to visualize time-to-event data, and group differences were assessed using log-rank tests. Before multivariable modeling, all predictor variables were assessed for multicollinearity using variance inflation factors (VIFs); a VIF > 5 indicated potential collinearity warranting further review or exclusion (Table 3). We applied natural log transformation to biomarkers with right-skewed distributions, including WBCNeut, TotalWBC, Monocytes, and NLR, before inclusion in Cox proportional hazards models (Supplementary Figure S1). Histograms and Shapiro–Wilk tests confirmed non-normality, and log transformation reduced the influence of extreme values, stabilized variance, and facilitated interpretation of hazard ratios as proportional changes, while preserving clinically meaningful heterogeneity in inflammatory responses among critically ill patients. Cox proportional hazards models were then used to identify biomarkers and demographic factors independently associated with survival. The proportional hazards assumption was tested for all covariates using Schoenfeld residuals and graphical diagnostics. A final Cox regression model was constructed, adjusting for potential confounders and including variables selected based on clinical relevance and statistical significance from univariate analyses. The final model was based on model-building processes (Supplementary Table S1). Hazard ratios (HRs) with 95% confidence intervals (CIs) were reported to quantify the strength and direction of associations between predictors and mortality risk. All analyses were conducted using R version 4.5.1 (R Foundation for Statistical Computing, Vienna, Austria) within the RStudio environment (RStudio PBC, Boston, MA, USA).

### Model-building process

The model-building process was performed to assess the robustness of model findings to different specifications of inflammatory markers and model complexity (Table 1). Given the clinical importance of neutrophil count (WBCNeut) in sepsis, we compared models using WBCNeut versus total white blood cell count. Additional models evaluated the inclusion or exclusion of PCT, monocytes, and the NRL to determine their influence on estimated hazard ratios. We also performed a focused comparison of 3 sequential models: (i) NLR with covariates but excluding neutrophils; (ii) neutrophils with covariates but excluding NLR; and (iii) a combined model including both NLR and neutrophils. Results for these comparisons are presented in Supplementary Table S1, with model performance quantified by Akaike Information Criterion (AIC), log-likelihood, and area under the curve (AUC) values. To further explore short-term discriminatory performance, time-dependent receiver operating characteristic (ROC) analyses were conducted at days 6, 10, and 15. These time points were selected as they consistently showed stronger discriminative performance. From a clinical perspective, they correspond to the end of the first week, mid-second week, and end of the second week of ICU admission, periods that typically coincide with patient reassessment in critical care. This strategy enabled us to assess model discrimination at clinically relevant intervals during the early phase of ICU admission, without suggesting these are standard decision points. Model performance was quantified using AUC values, with higher AUCs indicating better discrimination. In particular, Models 9 and 10, which incorporated full clinical and biomarker information (including WBCNeut and TotalWBC, respectively), demonstrated consistently higher AUCs (approximately 68–71%) compared to simpler models. ROC curves were used to visualize the trade-off between sensitivity and specificity at different thresholds, providing insight into each model’s ability to distinguish high-risk patients during the early phase of follow-up.

**Table 1:** Building process models

Model no.	Covariates	Description
1	Sex age group	Minimal model (baseline without lab markers)
2	Sex age group PCT	PCT only, to assess if PCT is predictive alone
3	Sex age group WBCNeut	Neutrophils only, to assess WBCNeut predictive value
4	Sex age group TotalWBC	Total WBC only, to assess predictive value of total WBC count
5	Sex age group PCT WBCNeut	PCT + WBCNeut, joint contribution of specific markers
6	Sex age group PCT TotalWBC	PCT + Total WBC, to compare to model 5, total vs neutrophils
7	Sex, age group, PCT, NLR, Monocytes	Assessing predictive value of NLR without neutrophils
8	Sex, age group, PCT, Neutrophils, Monocytes	Assessing predictive value of neutrophils without NLR
9	Sex age group PCT WBCNeut Monocytes NLR	Full model with WBCNeut, monocytes, and NLR
10	Sex age group PCT TotalWBC Monocytes NLR	Full model with TotalWBC, monocytes, and NLR

### Ethical considerations

Ethical approval was obtained from the Institutional Review Board of the University of Rwanda with reference number CMHS/IRB/042/2025. Permission to conduct the study was obtained from the administration of CHUB and RMRTH. Written informed consent was obtained from all study participants or their relatives before enrollment and data collection. Participants were free to opt out of the study at any stage during the study without loss of treatment privileges.

## Results

A total of 125 patients were followed up over 40 days. Of these, 56 (44.8%) were females and 67 (53.6%) were males. The proportion of deaths was marginally higher among males (56.7%) compared to females (50.0%), although this difference did not reach statistical significance (*p* = 0.453). The median age of surviving (censored) patients was 41 years (interquartile range (IQR): 30–58), compared to 50 years (IQR: 34–66) among those who died (*p* = 0.097). No statistically significant differences in survival status were observed across age groups (*p* = 0.520).

In univariate comparisons, patients who died exhibited higher median PCT levels (3.2 ng/ml, IQR: 0.7–24.6) compared to survivors (0.5 ng/ml, IQR: 0.2–1.3; *p* < 0.001), along with elevated neutrophil counts (11.5 vs. 5.3 × 10^3^/µL; *p* < 0.001), total WBC counts (13.5 vs. 8.0 × 10^3^/µL; *p* < 0.001), and NLR(12.0 vs. 4.0; *p* < 0.001). No statistically significant differences were observed in basophil (*p* = 0.491), monocyte (*p* = 0.219), or lymphocyte counts (*p* = 0.084) (Table 2).

**Table 2:** Sociodemographic and clinical characteristics of patients followed for 40 days

Variable	Category	No. of patients	Survival status (*n* = 125)		*p*-value
			Censored (%) *n* = 58	Deceased (%) *n* = 67	
Sex, *n* (%)	Female	56	29 (50.0)	29 (50.0)	0.453
	Male	67	29 (43.3)	38 (56.7)	
Age (years)	Median (IQR)	125	41 (30–58)	50 (34–66)	0.097
Age group (years)	Young adults (18–25)	17	10 (58.8)	7 (41.2)	0.520
	Middle-aged adults (26–40)	35	18 (51.4)	17 (48.6)	
	Older adults (41–55)	30	12 (40.0)	18 (60.0)	
	Elderly (56 and above)	43	18 (41.9)	25 (58.1)	
PCT levels (ng/ml)	Median (IQR)	125	0.5 (0.2–1.3)	3.2 (0.7–24.6)	< 0.001
Total WBCs × 10⁹/L	Median (IQR)	125	8.0 (5.1–12.2)	13.5 (9.0–25.1)	< 0.001
Neutrophils × 10⁹/L	Median (IQR)	125	5.3 (2.8–8.3)	11.5 (5.7–20.3)	< 0.001
Basophils × 10⁹/L	Median (IQR)	125	0.03 (0.02–0.05)	0.04 (0.02–0.08)	0.491
Eosinophils × 10⁹/L	Median (IQR)	125	0.04 (0.02–0.12)	0.03 (0.00–0.08)	0.058
Monocytes (µL) × 10⁹/L	Median (IQR)	125	0.72 (0.41–0.96)	0.91 (0.52–1.94)	0.219
Lymphocytes (µL) × 10⁹/L	Median (IQR)	125	1.23 (0.88–1.99)	0.94 (0.45–1.85)	0.084
NLR	Median (IQR)	125	4.0 (2.3–6.1)	12.0 (6.1–22.8)	< 0.001

### ICU sepsis survival estimates

**Fig. 1:**
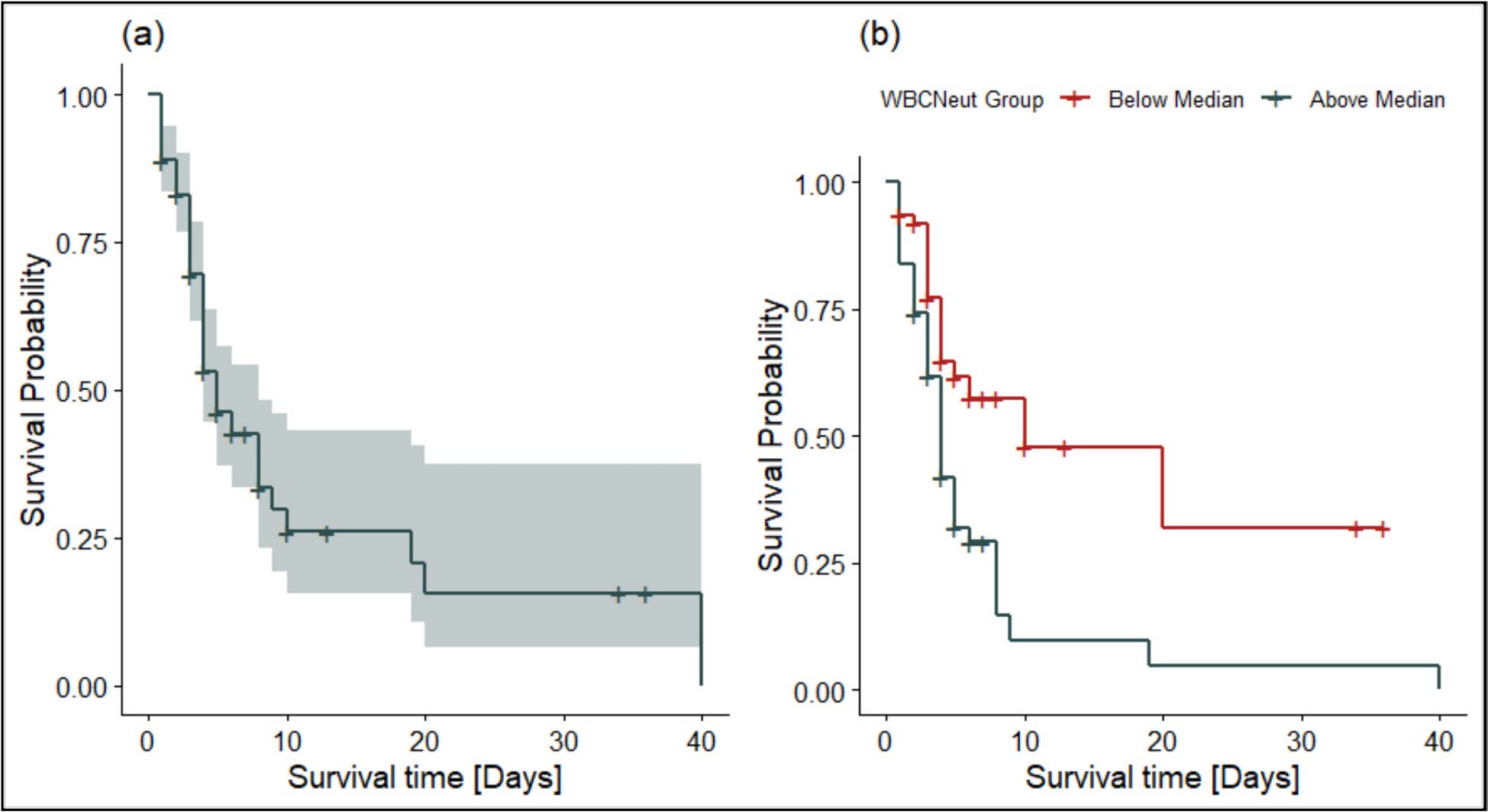
Kaplan–Meier survival curves for ICU patients with sepsis: **a** Overall Kaplan–Meier survival estimate shows that survival probability declined sharply within the first 10 days, indicating a high early mortality risk. **b** Kaplan–Meier survival estimates stratified by neutrophil count show that patients with higher neutrophil counts experience a steeper early decline in survival, indicating an increased risk of early mortality

The Kaplan–Meier survival curve (Fig. 1a) depicts the probability of survival over time among ICU patients diagnosed with sepsis. Survival probability declined sharply within the first 10 days, indicating a high early mortality risk. Beyond day 10, the survival curve plateaued, suggesting that patients who survived the initial critical phase faced a reduced subsequent risk of death. By day 20, the estimated survival probability was approximately 25%. The 95% confidence intervals widened at later time points due to the diminishing number of patients at risk. In Fig. 1b, the survival curves demonstrate notable differences between neutrophil strata. Although the survival curve for patients with neutrophil counts below the median initially dipped below 100% due to early adverse events, death within 24 h of enrollment, these individuals demonstrated consistently higher survival probabilities throughout the follow-up period. In contrast, patients with higher neutrophil counts showed a steeper early decline in survival, indicating increased early mortality risk.

### Multicollinearity assessment

We assessed multicollinearity among covariates using variance inflation factors. All VIFs were below 2.5 (mean VIF = 1.85), indicating no evidence of problematic collinearity (Table 3). The highest VIF was observed for the elderly age group (VIF = 2.41), which remained within acceptable thresholds. Moderate collinearity was noted between TotalWBC and WBCNeut. Given the stronger biological plausibility and greater clinical specificity of neutrophil count as an infection- and sepsis-related marker, WBCNeut was retained in the primary Cox regression models. To account for the potential contribution of overall leukocyte burden, TotalWBC was evaluated in separate models as part of the sensitivity analyses, where it replaced WBCNeut in otherwise equivalent model specifications.

**Table 3:** VIF for Cox model predictors of survival

Variable	VIF	1/VIF
Procalcitonin	1.50	0.667
Neutrophils	1.83	0.547
Monocytes	1.55	0.644
NLR	1.83	0.546
Sex—male	1.08	0.923
Age group: middle-aged adults	2.30	0.435
Age group: older adults	2.29	0.436
Age group: elderly	2.41	0.415
Mean VIF	1.85	

### Proportional hazards assumption test

We assessed the proportional hazards assumption using Schoenfeld residuals for each covariate and globally. None of the individual variables, including sex, age group, PCT, neutrophil count (WBCNeut), monocytes, or NLR, showed significant deviation from proportionality (all *p* > 0.05). The global test (*χ*^2^ = 4.23, df = 8, *p* = 0.836) indicated no evidence of violation of the proportional hazards assumption for the model as a whole.

### Time-dependent discrimination of predictive models for ICU mortality

**Fig. 2:**
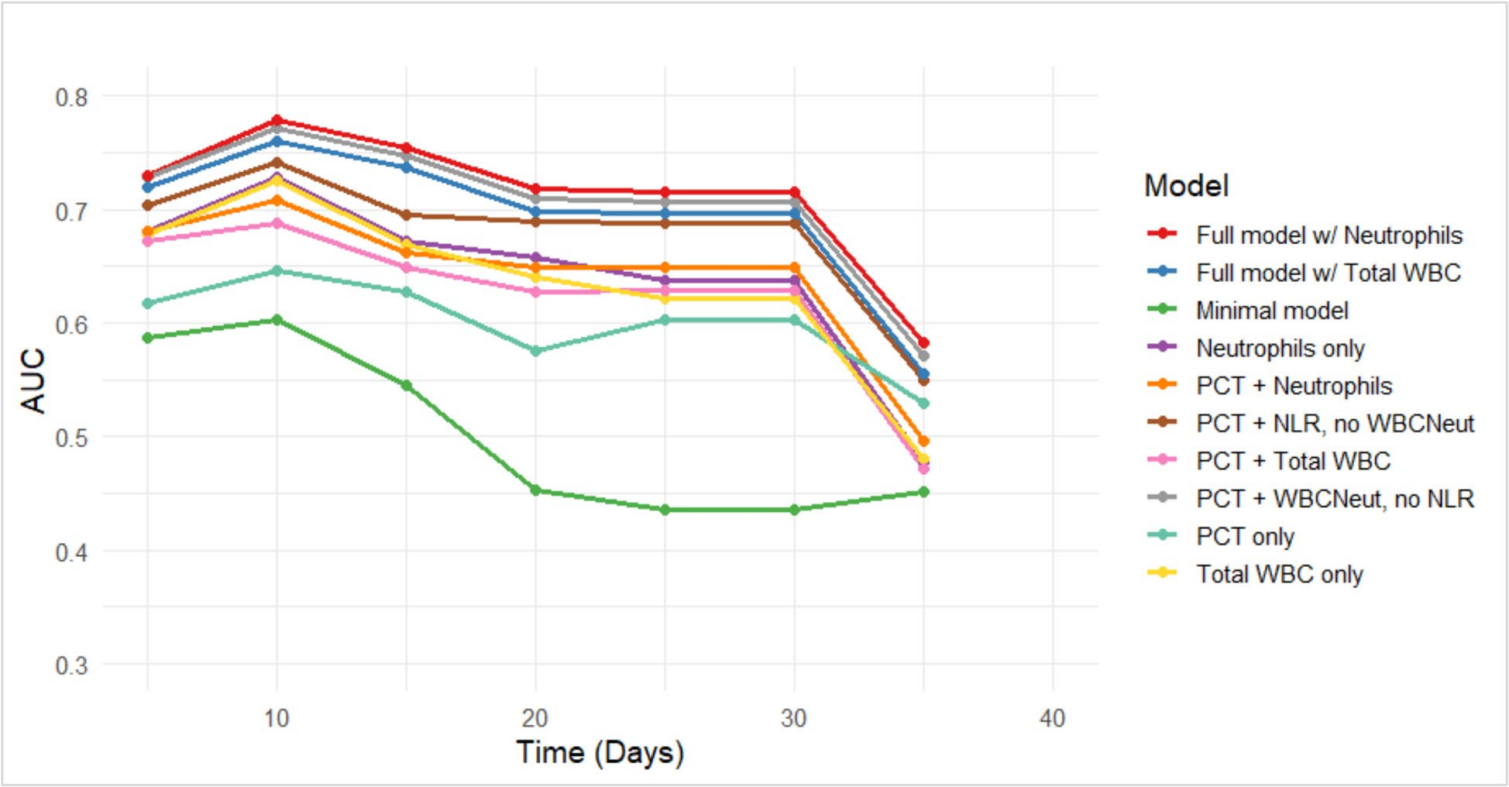
Time-dependent AUC curves for eight Cox models predicting ICU mortality over 40 days

Time-dependent AUCs were constructed to assess and compare the discriminative performance of ten multivariable Cox models (all adjusted to age and sex) across the 40-day follow-up period. As illustrated in Fig. 2 (also Supplementary Table S1), full models incorporating both clinical variables and either neutrophil count or total WBC count consistently yielded the highest and most stable AUCs, particularly during the first 10 days (AUC ~ 0.76–0.78), followed by a gradual decline thereafter. The minimal model, which excluded biomarker data, exhibited the weakest performance throughout, with AUCs falling below 0.5 after day 20. Models combining PCT with neutrophils or WBC outperformed single-biomarker or PCT-only models, especially in early follow-up. The overall decline in AUCs over time likely reflects the waning predictive value of baseline measurements and increasing variability in clinical outcomes.

### ROC curve analysis of sepsis biomarkers

**Fig. 3:**
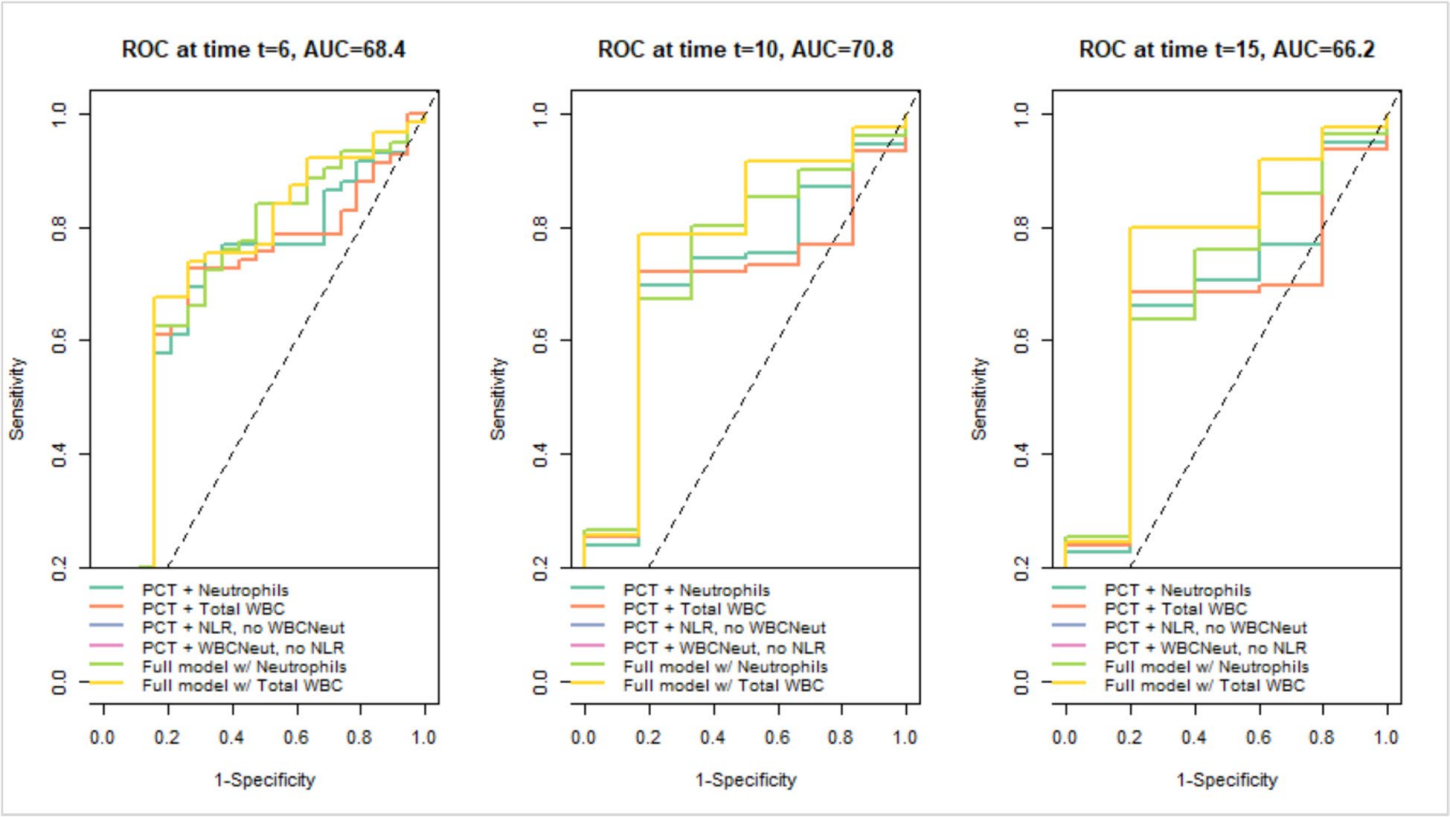
ROC curves showing sensitivity vs. specificity for Models 5–10 at days 6, 10, and 15

To evaluate short-term predictive accuracy, ROC curves for Models 5–10 were evaluated at days 6, 10, and 15. The time-dependent AUCs reflect the probability of death by each time point, derived from Cox models predicting ICU mortality over a 40-day follow-up period (Fig. 2). Multiple intervals were explored, and these specific points were chosen because they consistently demonstrated higher discriminative performance. Clinically, they correspond to approximately the end of the first week, mid-second week, and end of the second week of ICU admission, periods that often reflect patient reassessment in critical care. This approach allowed for the evaluation of model discrimination at time intervals that are meaningful for tracking changes in patient risk over the early course of ICU admission, without implying these are standardized decision points. The analysis showed that Models 9 and 10, incorporating clinical data plus neutrophil count (Model 9) or total WBC count (Model 10), consistently outperformed simpler models. At days 6 and 10, both achieved AUCs of 68–71%, indicating good short-term discriminative performance. Although predictive accuracy declined slightly by day 15 (AUC ~ 66%), these models remained comparatively stable, highlighting the added value of integrating multiple biomarkers for early sepsis mortality risk stratification (Fig. 3). A sensitivity analysis of Cox regression models using various biomarker combinations is provided in Supplementary Table S1.

### Predictors of ICU mortality in sepsis

In the final multivariable Cox regression model 5, elevated neutrophil counts emerged as the only independent predictor of mortality among ICU patients (Table 4). After adjusting for key demographic and biomarker covariates, higher neutrophil counts were strongly associated with increased ICU mortality. Specifically, a ~threefold increase in neutrophil count was associated with a twofold higher hazard of death [aHR (adjusted hazard ratio) = 1.99, 95% CI 1.37–2.88; *p* < 0.001]. This finding demonstrates the prognostic significance of neutrophil count as a marker of poor clinical outcome. In contrast, no statistically significant associations were observed between survival and sex, age group, and procalcitonin levels, suggesting that these factors may have limited independent predictive value for short-term mortality when included alongside neutrophil count in a multivariable mode (Table 4).

**Table 4:** Biomarker and demographic factors associated with survival in ICU patients

Variable	cHR (95% CI)	aHR (95% CI)
Sex		
Female	Ref	Ref
Male	1.19 (0.71, 2.01)	1.34 (0.79, 2.27)
Age (years)	1.01 (0.99, 1.02)	1,01 (1.00, 1.02)
Procalcitonin	1.17 (1.05, 1.31)**	1.11 (0.99, 1.25)
Neutrophils	2.14 (1.49, 3.06) ***	1.99 (1.37, 2.88)***

95% confidence intervals in brackets

^*^ *p* < 0.05, ** *p* < 0.01, *** *p* < 0.001

### Model-building process

We conducted a sensitivity analysis to evaluate the robustness and consistency of Cox regression models under varying combinations of covariates, with a particular focus on WBC markers. All models included sex and age group as adjustment variables; however, these demographic covariates consistently showed non-significant associations with survival, suggesting limited predictive utility in this context.

In contrast, models incorporating neutrophil count (WBCNeut), NLR, or total white blood cell count (total WBC) demonstrated statistically significant positive associations with increased hazard of death, indicating that elevated levels of these markers may serve as useful indicators of poor prognosis. Specifically, WBCNeut was significantly associated with mortality in Models 3, 5, and 9, while total WBC showed similar associations in Models 4 and 6, and NLR in Model 7.

Combination models (Models 5–10), which included Procalcitonin (PCT) alongside WBCNeut, NLR, or total WBC, generally achieved better model fit, as reflected in higher log-likelihood values (ranging from − 226.349 to − 230.581). Models 9 and 10 demonstrated the strongest predictive performance, consistent with ROC curve analysis showing higher AUC values (~ 68–71%), indicating moderate-to-good discrimination. Although Model 7 (log-likelihood = − 226.787) fit the data slightly better, Model 5 (AIC = 467.367; log-likelihood = − 227.684) was selected for the final analysis due to its lower AIC and more consistent, interpretable predictor estimates, providing a robust model. Importantly, the key findings regarding WBC-related markers were consistent across models, supporting the robustness and reliability of the results.

## Discussion

This prospective cohort study presents critical insights into the prognostic utility of PCT and WBC parameters—particularly the neutrophil count and NLR—in adult patients admitted to the ICU with suspected sepsis in Rwanda. With an overall mortality rate of 53.6% and with 8.8% of patients dying within 24 h of admission, our findings underscore the acute lethality of sepsis and the pressing need for timely risk stratification, especially in resource-constrained settings [2, 15]. The significant elevation of serum PCT levels in non-survivors (median 4.45 ng/mL), particularly those who died within 24 h (median 2.2 ng/mL), reinforces its established role as a reliable biomarker of sepsis severity [16–18]. These results align with existing literature indicating that procalcitonin (PCT) levels exceeding 2 ng/mL are associated with a heightened inflammatory response and an increased risk of mortality in patients with severe bacterial infections and sepsis [19–21].

While PCT alone did not retain statistical significance in adjusted survival models, its association with poor outcomes suggests it remains a clinically valuable early warning marker. Complementing PCT, the NLR demonstrated strong discriminative ability, with fatal cases exhibiting a median ratio of 12.47 compared to 4.02 among survivors (*p* < 0.001). Elevated neutrophil counts were independently associated with increased mortality (aHR = 1.99; 95% CI 1.37–2.88; *p* = 0.001), confirming their mortality predictive value even after adjusting for age and sex. This finding aligns with a study conducted at a rural district hospital in Rwanda that reported female gender and leukocytosis as significant risk factors for sepsis-related mortality [8]. Notably, preserved lymphocyte counts among non-survivors in this cohort contrast with immunosuppressive profiles reported in other populations, such as the persistent lymphopenia observed by Drewry et al. as a predictor of mortality in septic patients in the United States [22]. This divergence may reflect differences in patient characteristics, comorbidities, or health system factors, and should be interpreted cautiously. While regional variation in immune response is a possible explanation [23, 24], our exclusion of advanced HIV/AIDS patients—a group more likely to present with lymphopenia—limits direct comparison. Further studies, including broader patient populations, are needed to clarify these patterns.

The Kaplan–Meier survival curve further emphasizes the critical nature of early sepsis progression, with the steepest decline in survival observed within the first 10 days post-admission. Patients who survived beyond this threshold experienced a markedly lower risk of mortality. By day 20, the cumulative survival probability had declined to approximately 25%, reflecting the high early attrition in this cohort. Cox proportional hazards modeling revealed that among all variables considered, neutrophil count remained a statistically robust independent predictor of death. Importantly, neither age group nor sex emerged as significant survival predictors, despite a trend toward increased early mortality among elderly patients (≥ 56 years). The absence of significant multicollinearity and adherence to the proportional hazards assumption further supports the internal validity of these survival models. Sensitivity analyses confirmed that combinations of PCT with neutrophil or total WBC counts enhanced model performance, with Model 7 (incorporating PCT, neutrophils, monocytes, and NLR) demonstrating the best fit (log-likelihood = − 227.29). These findings suggest the synergistic utility of biomarker panels in survival prediction and risk stratification.

Given the limitations in diagnostic infrastructure and ICU bed capacity in Rwanda, the application of cost-effective biomarkers like neutrophil and NLR could transform sepsis management. Their routine use on ICU admission may help identify patients requiring escalated care, prompt antibiotic initiation, and prioritization of supportive therapies. The integration of these markers into triage protocols and context-specific scoring systems, such as the Kigali Surgical Sepsis Score, [25], and additional clinical data such as vital signs and comorbidities would further enhance clinical utility. The observed phenotype of rapid clinical deterioration—with extreme biomarker elevations (PCT > 24 ng/mL, NLR > 28) and early mortality—could define a critical subgroup warranting aggressive early management. Importantly, the immune preservation seen in lymphocyte counts among non-survivors invites further exploration into the unique immunopathology of sepsis in African populations.

## Strengths and limitations

This study has several notable strengths. Its prospective design allowed for the capture of real-world ICU dynamics, minimizing recall bias and providing a more accurate representation of patient outcomes. The research also addresses a critical knowledge gap in sepsis biomarker research within sub-Saharan Africa, making the findings highly relevant to the local context. Furthermore, the joint evaluation of PCT and WBC parameters enhanced diagnostic precision and prognostic value, highlighting the potential benefits of biomarker synergy. Despite these strengths, the study has some limitations. The final sample size of 125 patients fell short of initial estimates, which may have affected the detection of more nuanced associations. Additionally, the study's reliance on single-time-point biomarker evaluation precluded temporal trend analysis, which could have enhanced the understanding of dynamic immune responses. The unavailability of SOFA or qSOFA scores limited comparative risk stratification, and the exclusion of immunocompromised individuals may have affected the generalizability of the findings to broader ICU populations.

## Conclusion

This study concludes that elevated PCT and NLR values are associated with mortality in sepsis patients in Rwandan ICUs. Specifically, PCT > 2 ng/mL and NLR > 10 were identified as threshold values for early risk identification, while patients with PCT > 24 ng/mL and NLR > 28 were found to be at high risk of rapid deterioration. The study suggests that integrating these biomarkers into ICU practice could improve patient outcomes in resource-limited settings. Future studies should focus on validating these findings through multicenter collaborations across diverse African settings, conducting longitudinal biomarker monitoring to define prognostic windows, and integrating biomarker evaluation with sepsis scoring systems to refine predictive accuracy. Additionally, investigating immune phenotypes in fatal cases without lymphopenia may provide novel insights into sepsis mechanisms. By pursuing these directions, future research can further enhance our understanding of sepsis biomarkers and improve patient care, sepsis management protocols, and reduce mortality rates in high-burden, resource-constrained environments.

## Supplementary Information

Supplementary Material 1.

Supplementary Material 2.

## Data Availability

All data generated in this study are included in this manuscript. However, the dataset will be made available by the corresponding author upon reasonable request.

## Abbreviations

ICU: Intensive care unit
PCT: Procalcitonin
WBC: White blood cell
NLR: Neutrophil-to-lymphocyte ratio
CHUB: Butare University Teaching Hospital
RMRTH: Rwanda Military Referral and Teaching Hospital
CBC: Complete blood count
EDTA: Ethylene diamine tetraacetic acid
ROC: Receiver operating characteristic
AUC: Area under the curve
VIF: Variance inflation factor
HR: Hazard ratio
CI: Confidence interval
SOFA: Sequential Organ Failure Assessment
qSOFA: Quick Sequential Organ Failure Assessment
