# Supplementary material for: Prognostic value of low-cost white blood cell indices and procalcitonin for mortality in Rwandan sepsis patients: a prospective intensive care unit study
Source: Trop Med Health. 2025 Oct 9;53:135. doi: 10.1186/s41182-025-00815-4 (PMC12509366; doi:10.1186/s41182-025-00815-4)
Supplement: Supplementary file 1 — Supplementary Material 1. [file 41182_2025_815_MOESM1_ESM.docx]

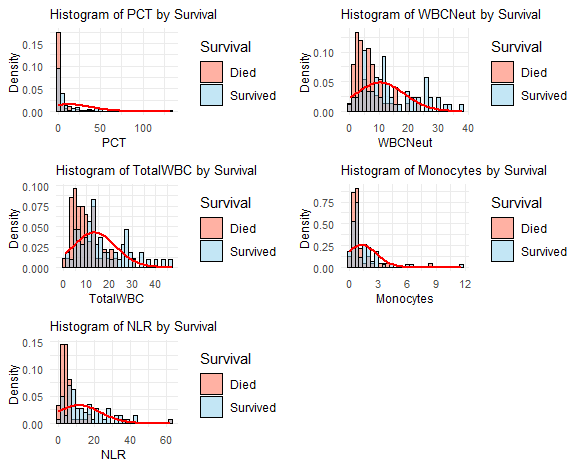


**Figure S1: Normality check**

Histograms of WBCNeut, TotalWBC, PCT, Monocytes, and NLR revealed right-skewed distributions, with most patients exhibiting lower values and a few extreme high values. Overlaid normal density curves poorly fit the data, consistent with Shapiro–Wilk test results indicating non-normality. To account for this skewness and stabilize variance, we applied a natural logarithm transformation to these biomarkers before inclusion in Cox proportional hazards models. While Cox models do not require predictors to be normally distributed, log transformation improves interpretability by reducing the influence of extreme values and allows hazard ratios to reflect proportional changes in biomarker levels.
