# Supplementary material for: Prognostic value of low-cost white blood cell indices and procalcitonin for mortality in Rwandan sepsis patients: a prospective intensive care unit study
Source: Trop Med Health. 2025 Oct 9;53:135. doi: 10.1186/s41182-025-00815-4 (PMC12509366; doi:10.1186/s41182-025-00815-4)
Supplement: Supplementary file 2 — Supplementary Material 2. [file 41182_2025_815_MOESM2_ESM.rtf]

Table S1: Cox regression model building processes (Coefficients / Log HRs)
	(1)	(2)	(3)	(4)	(5)	(6)	(7)	(8)	(9)	(10)	
	_t	_t	_t	_t	_t	_t	_t	_t	_t	_t	
1.Sex - Female	ref	ref	ref	ref	ref	ref	ref	ref	ref	ref	
											
2.Sex - male	0.195	0.216	0.262	0.293	0.300	0.320	0.167	0.270	0.202	0.223	
	[-0.336,0.727]	[-0.316,0.748]	[-0.272,0.797]	[-0.239,0.826]	[-0.239,0.840]	[-0.217,0.857]	[-0.377,0.711]	[-0.274,0.814]	[-0.362,0.766]	[-0.334,0.781]	
											
1. Young Adults (18–25)	ref	ref	ref	ref	ref	ref	ref	ref	ref	ref	
											
2. Middle-aged Adults (26–40)	0.042	-0.095	0.474	0.364	0.364	0.273	0.429	0.391	0.428	0.422	
	[-0.865,0.950]	[-1.014,0.825]	[-0.445,1.392]	[-0.552,1.279]	[-0.570,1.297]	[-0.655,1.202]	[-0.514,1.373]	[-0.549,1.330]	[-0.515,1.371]	[-0.522,1.366]	
											
3. Older Adults (41–55)	0.179	-0.090	0.596	0.485	0.399	0.284	0.446	0.424	0.454	0.452	
	[-0.728,1.086]	[-1.018,0.839]	[-0.330,1.523]	[-0.432,1.401]	[-0.559,1.357]	[-0.662,1.230]	[-0.528,1.419]	[-0.540,1.388]	[-0.518,1.425]	[-0.519,1.424]	
											
4. Elderly (56 and above)	0.282	0.210	0.531	0.458	0.508	0.438	0.605	0.539	0.592	0.594	
	[-0.589,1.153]	[-0.659,1.078]	[-0.345,1.406]	[-0.416,1.332]	[-0.369,1.385]	[-0.437,1.313]	[-0.285,1.496]	[-0.341,1.420]	[-0.299,1.483]	[-0.296,1.483]	
											
PCT		0.176**			0.101	0.109	-0.092	0.137	-0.019	0.047	
		[0.058,0.294]			[-0.021,0.223]	[-0.013,0.232]	[-0.284,0.099]	[-0.001,0.276]	[-0.386,0.349]	[-0.300,0.395]	
											
WBCNeut			0.810***		0.697***			0.756***	0.268		
			[0.440,1.180]		[0.316,1.079]			[0.356,1.157]	[-0.873,1.409]		
											
TotalWBC				0.879***		0.741**				0.586	
				[0.447,1.311]		[0.297,1.184]				[-0.628,1.799]	
											
Monocytes							0.506*	-0.207	0.269	-0.005	
							[0.052,0.959]	[-0.573,0.160]	[-0.837,1.374]	[-1.149,1.139]	
											
NLR							0.757***		0.510	0.352	
							[0.369,1.144]		[-0.609,1.628]	[-0.556,1.260]	
Log-likelihood	-239.272	-234.930	-229.016	-230.581	-227.684	-229.032	-226.787	-227.079	-226.681	-226.349	
N	112.000	112.000	112.000	112.000	112.000	112.000	112.000	112.000	112.000	112.000	
AIC	486.544	479.861	468.032	471.162	467.367	470.063	467.574	468.157	469.361	468.698	
AUC, 6 Days	0.607	0.634	0.683	0.676	0.684	0.676	0.707	0.724	0.728	0.712	
AUC, 10 Days	0.602	0.646	0.728	0.725	0.708	0.688	0.742	0.772	0.779	0.760	
AUC, 15 Days	0.545	0.627	0.672	0.669	0.662	0.649	0.695	0.747	0.754	0.737	
Key: NLR - Neutrophil to lymphocyte ratio (NLR) 
95% confidence intervals in brackets
* p < 0.05, ** p < 0.01, *** p < 0.001

We conducted a sensitivity analysis to evaluate the robustness and consistency of Cox regression models under varying combinations of covariates, with a particular focus on WBC markers. All models included sex and age group as adjustment variables; however, these demographic covariates consistently showed non-significant associations with survival, suggesting limited predictive utility in this context. 

In contrast, models incorporating neutrophil count (WBCNeut), Neutrophil to lymphocyte ratio (NLR) or total white blood cell count (total WBC) demonstrated statistically significant positive associations with increased hazard of death, indicating that elevated levels of these markers may serve as useful indicators of poor prognosis. Specifically, WBCNeut was significantly associated with mortality in Models 3, 5 and 9, while total WBC showed similar associations in Models 4 and 6 and Neutrophil to lymphocyte ratio (NLR) in model 7.

Combination models (Models 5–10), which included Procalcitonin (PCT) alongside WBCNeut, NLR or total WBC, generally achieved better model fit, as reflected in higher log-likelihood values (ranging from −226.349 to −230.581). Models 9 and 10 demonstrated the strongest predictive performance, consistent with ROC curve analysis showing higher AUC values (~68–71%), indicating moderate-to-good discrimination. Although Model 7 (log-likelihood = -226.787) fit the data slightly better, Model 5 (AIC = 467.367; log-likelihood = -227.684) was selected for the final analysis due to its lower AIC and more consistent, interpretable predictor estimates, providing a parsimonious and robust model. Importantly, the key findings regarding WBC-related markers were consistent across models, supporting the robustness and reliability of the results.
